# MECHANISM OF ACTION: AGE DIFFERENCES IN RESPONSE TO ACCEPTANCE AND COMMITMENT THERAPY FOR CHRONIC PAIN

**DOI:** 10.1093/geroni/igz038.2979

**Published:** 2019-11-08

**Authors:** Julie L Wetherell, Matthew Herbert, Niloofar Afari

**Affiliations:** VA San Diego Healthcare System, San Diego, California, United States

## Abstract

A recent randomized comparison of Acceptance and Commitment Therapy (ACT) vs. Cognitive-Behavioral Therapy for chronic pain found a clear age interaction effect, such that older adults benefitted more from ACT. In a subsequent study comparing ACT delivered in person to ACT delivered via telehealth to a sample of veterans (N=128, mean age 51.9, SD 13.3, range 25-89), we found no significant age by modality interactions, suggesting that older veterans responded as well as younger people did to telehealth delivery. Consistent with our previous findings, we found a trend for older adults to experience greater reduction in pain interference (p = .051) and significantly greater reduction in pain severity (p = .001) than younger adults following ACT. In younger veterans, change in pain acceptance from baseline to posttreatment was related to change in pain interference from baseline to 6-month follow-up (r = -.38), but change in pain interference from baseline to posttreatment was not related to change in pain acceptance from baseline to follow-up (r = .14), suggesting that, consistent with the ACT model, increased pain acceptance at posttreatment was related to reduced pain interference at follow-up. By contrast, in older veterans, both correlations were significant and of comparable magnitude (rs = -.43 and -.46, respectively), providing no support for the idea that change in pain acceptance drove change in pain interference. Overall, our findings suggest that ACT may work better in older adults with chronic pain than in younger adults, but via a different mechanism.

